# Commentary: Synergistic treatment of sodium propionate and Sishen Pill for diarrhea mice with kidney-yang deficiency syndrome

**DOI:** 10.3389/fcimb.2025.1641826

**Published:** 2025-08-22

**Authors:** Zilin Zhao, Hejia Wan, Xiaohui Liu

**Affiliations:** ^1^ School of Nursing (Nursing School of Smart Healthcare Industry), Henan University of Chinese Medicine, Zhengzhou, China; ^2^ Henan Provincial Hospital of Traditional Chinese Medicine (The Second Affiliated Hospital of Henan University of Traditional Chinese Medicine), Zhengzhou, China

**Keywords:** kidney-yang deficiency, gut-kidney axis, microbial metabolites, Sishen Pill, intestinal barrier dysfunction, dysbiosis

## Introduction

Kidney-yang deficiency syndrome (KYDS), a fundamental concept in Traditional Chinese Medicine (TCM), manifests clinically as chronic diarrhea, cold intolerance, and fatigue with high prevalence among aging populations. Modern biomedical research has established that KYDS correlates with hypothalamic-pituitary-adrenal (HPA) axis dysregulation and intestinal barrier compromise (Zhang et al., 2019). Crucially, emerging evidence implicates gut dysbiosis as a pathological mediator, where depleted short-chain fatty acid (SCFA) production—particularly propionate—impairs colonic mucosal integrity and immune homeostasis (Smith et al., 2021). Sishen Pill (SSP), a classical TCM formula documented in Sheng Ji Zong Lu (1100 AD), contains bioactive compounds like psoralen and evodiamine that demonstrate anti-inflammatory and microbiota-modulating properties (Ge et al., 2020). The innovative integration of sodium propionate (SP) with SSP represents a paradigm-shifting strategy targeting both microbial ecology and mucosal healing. This synergy is theoretically anchored in TCM’s “kidney governing water metabolism” principle and Western medicine’s gut-kidney axis concept, offering unprecedented potential for refractory diarrhea management.

## General comments

In a recent study entitled “Synergistic Treatment of Sodium Propionate and Sishen Pill for Diarrhea Mice with Kidney-Yang Deficiency Syndrome” was published in Frontiers in Cellular and lnfection Microbiology”.Synergistic Treatment of Sodium Propionate and Sishen Pill for Diarrhea Mice with Kidney-Yang Deficiency Syndrome” (Guo et al., 2025) pioneers an integrative therapeutic strategy combining sodium propionate—a gut microbiota-modulating short-chain fatty acid—with Sishen Pill, a classic TCM formula, to address diarrhea in murine models of kidney-yang deficiency syndrome. The authors investigated the synergistic potential of metabolic regulation and TCM principles in restoring intestinal homeostasis. Key strengths include an innovative combination therapy design, comprehensive validation of TCM syndromes in animal models, and multi-faceted biomarker analysis spanning intestinal enzymes, immune organ indices, and microbial composition. Their systematic evaluation demonstrated robust synergistic efficacy in alleviating diarrhea and restoring microbiota balance. Critically, the 75% Sishen Pill + 60 mg/kg sodium propionate group exhibited normalization of fecal water content, bacterial overgrowth, and immune dysfunction, underscoring the importance of mechanistic synergy and precise dose optimization for therapeutic outcomes. This work offers novel insights into microbiota-TCM interactions and paves the way for optimized integrative interventions.

First, several limitations should be acknowledged. While the study identifies shifts in Bifidobacterium and Escherichia coli populations, the causal link between these changes and diarrhea resolution remains unclear. Functional assays, such as metabolomics profiling of short-chain fatty acids, could strengthen mechanistic interpretations (He et al., 2022). Additionally, the empirical determination of the optimal Sishen Pill-to-sodium propionate ratio lacks exploration of pharmacokinetic interactions or receptor-mediated signaling pathways, leaving the basis for this specific combination unexplained. The study’s exclusive focus on murine models also restricts immediate translational relevance, necessitating clinical validation to confirm safety and efficacy in humans.

Second, several mechanistic ambiguities warrant critical examination. While the reported microbial alterations offer correlative insights, the absence of functional validation leaves unresolved whether these taxonomic changes directly mediate therapeutic effects or merely represent secondary phenomena. Metabolomic profiling of luminal SCFA dynamics—particularly quantification of propionate flux and butyrate reciprocation—would substantially strengthen causal attribution, especially given butyrate’s established role as the primary colonocyte energy source (Anderson et al., 2022).

Furthermore, the empirically determined optimal ratio lacks pharmacokinetic substantiation regarding potential herb-metabolite interactions. Unanswered questions persist concerning whether bioactive SSP constituents such as psoralen competitively inhibit monocarboxylate transporter (MCT1)-mediated propionate absorption, or conversely potentiate GPR43 receptor signaling in enteroendocrine cells. The exclusive reliance on antibiotic-induced murine models also constrains clinical extrapolation, as human KYDS involves distinctive neuroendocrine signatures including cortisol rhythm disruption and adrenocortical insufficiency that remain inadequately recapitulated in rodents.

## Discussion

The groundbreaking work by Guo et al. inaugurates a transformative approach to KYDS management by synergizing ancient herbal wisdom with microbial metabolome modulation. Their demonstration that SP potentiates SSP’s efficacy aligns with contemporary understanding of the gut-kidney axis, where SCFAs directly influence renal water handling through olfactory receptor 78 (Olfr78) activation in juxtaglomerular apparatus. Nevertheless, three fundamental questions demand resolution to advance this therapeutic strategy. First, the causal relationship between specific microbial shifts and clinical improvement requires validation through fecal microbiota transplantation studies in germ-free models (He et al., 2019). Second, the pharmacokinetic interplay between SSP polyphenols and SP absorption necessitates investigation via Caco-2 transwell assays and hepatic microsomal stability testing (Zhang et al., 2015). Third, the neuroendocrine dimension of KYDS—particularly HPA axis modulation—remains unexplored; future research should assess corticosterone rhythms and adrenal glucocorticoid output in treated models.

1. SP serving as a histone deacetylase (HDAC) inhibitor, enhances SSP-induced Nrf2 activation. Existing evidence suggests that HDAC inhibition enhances negative feedback regulation by upregulating glucocorticoid receptor (GR) expression, and this process may be further amplified by gut-derived SCFAs (such as butyrate) through the GPR43-HDAC inhibition axis, exacerbating gut-kidney inflammation in Nrf2-deficient mice (Wang et al., 2012). Additionally, the renal protective effect of vagus nerve stimulation depends on the α7nAChR-Nrf2 pathway, suggesting that Nrf2 is a core target for neuro-immune regulation.
2. The gut microbiota is closely associated with KYDS (Chen et al., 2019). Braune (Braune, 2025) demonstrated that SSP isoflavones promote the conversion of propionic acid to butyric acid via bacteria expressing butyryl-CoA transferase.
3. The vagus nerve afferent fibers regulate colonic motility through the gut-brain axis. Muller et al (Muller et al., 2020)’s research revealed that the specific mechanism involves vagus nerve afferent fibers sensing intestinal signals via 5-HT_3_ receptors and SCFAs/GPR41/43, enhancing parasympathetic output through the NTS-DMV pathway, and promoting colonic peristalsis. Gut microbiota metabolites (butyrate, tryptophan) regulate this process through epigenetic regulation (HDAC-BDNF) and neurotransmitter regulation.

To validate these hypotheses, multi-omics integration is crucial—metabolomics sequencing combined with mucosal proteomics can reveal microbial functional pathways and host receptor responses. Crucially, clinical translation requires phased human trials: first, precise stratification based on traditional Chinese medicine syndromes (KYDS) combined with biomarkers (24-hour urinary cortisol, ACTH) and gut type (metagenomic typing); Phase I trials assess SP/SSP pharmacokinetics (blood-brain barrier permeability, TGF-β1 safety monitoring) to determine dosing regimens; Phase II adopts a randomized double-blind design, with the experimental group receiving SP/SSP combined with microbial-targeted intervention (e.g., Prevotella-type anti-inflammatory fiber supplementation), and the control group receiving a placebo. Primary endpoints include colon transit time (SmartPill), neuropeptide levels (ELISA/MS), and microbial function (metagenomics + metabolomics), secondary endpoints assess autonomic nervous function (heart rate variability) and psychological scores (HADS); ultimately, through multi-omics integration (mucosal proteomics-fecal metagenomics), the regulatory mechanisms of the HDAC3/Nrf2 pathway will be elucidated to provide evidence for personalized treatment.
